# Biogeographic Ancestry Is Associated with Higher Total Body Adiposity among African-American Females: The Boston Area Community Health Survey

**DOI:** 10.1371/journal.pone.0122808

**Published:** 2015-04-13

**Authors:** Sunali D. Goonesekera, Shona C. Fang, Rebecca S. Piccolo, Jose C. Florez, John B. McKinlay

**Affiliations:** 1 Department of Epidemiology and Biostatistics, New England Research Institutes, 480 Pleasant St., Watertown, MA 02472, United States of America; 2 Department of Environmental Health, Harvard School of Public Health, Boston, MA 02115, United States of America; 3 Diabetes Unit/ Center for Human Genetic Research, Massachusetts General Hospital, Boston, MA 02114, United States of America; The University of Texas Health Science Center (UTHSCSA), UNITED STATES

## Abstract

**Objectives:**

The prevalence of obesity is disproportionately higher among African-Americans and Hispanics as compared to whites. We investigated the role of biogeographic ancestry (BGA) on adiposity and changes in adiposity in the Boston Area Community Health Survey.

**Methods:**

We evaluated associations between BGA, assessed via Ancestry Informative Markers, and adiposity (body mass index (BMI), percent body fat (PBF), and waist-to-hip ratio (WHR)) and changes in adiposity over 7 years for BMI and WHR and 2.5 years for PBF, per 10% greater proportion of BGA using multivariable linear regression. We also examined effect-modification by demographic and socio-behavioral variables.

**Results:**

We observed positive associations between West-African ancestry and cross-sectional BMI (percent difference=0.62%; 95% CI: 0.04%, 1.20%), and PBF (β=0.35; 95% CI: 0.11, 0.58). We also observed significant effect-modification of the association between West-African ancestry and BMI by gender (*p*-interaction: <0.002) with a substantially greater association in women. We observed no main associations between Native-American ancestry and adiposity but observed significant effect-modification of the association with BMI by diet (*p*-interaction: <0.003) with inverse associations among participants with higher Healthy Eating Scores. No associations were observed between BGA and changes in adiposity over time.

**Conclusion:**

Findings support that West-African ancestry may contribute to high prevalence of total body adiposity among African-Americans, particularly African-American women.

## Introduction

Obesity is a major public health concern which significantly increases one’s risk for adverse health including type II diabetes, cardiovascular disease, musculoskeletal disorders [1] and depression [2]. The prevalence of obesity is substantially higher among African-Americans and Hispanics as compared to non-Hispanic whites in the United States [3]. Moreover, a study performed on the National Health and Nutrition Examination Survey (NHANES) data from the last two decades has found a widening of racial disparities in regard to body mass index (BMI) and waist circumference (WC) between non-Hispanic whites and African-Americans, and has estimated the prevalence of central obesity and obesity among black women in 2020 to reach 90.9 and 70.7%, respectively [4]. The racial/ethnic differences in obesity and related diseases persist in specific populations even after adjusting for socio-economic variables [5–8] indicating that genetics may independently contribute to racial/ethnic disparities in obesity.

The genetic basis for racial/ethnic disparities in adiposity and metabolic disease could be explained by the thrifty gene hypothesis which was first proposed in 1960’s [9, 10]. According o this theory, genes that that promote the storage of energy as body fat, that may have been advantageous during Paleolithic times, may lead to obesity and metabolic disease in modern western societies where there is little need for fat storage. It has been hypothesized that “thrifty genes” may be more prevalent among racial/ethnic subgroups outside of those of European descent as many of these individuals have ancestral origins in regions where droughts and famine commonly occur. Even though this theory has received some criticism [11], multiple studies have since evaluated associations between genetic ancestry and adiposity-associated diseases in western societies.

Ancestral Informative Markers (AIMs), i.e., genetic markers unique to people of a homogenous BGA, are often used as correlates of self-identified race in studies performed in admixed populations [5–7, 12–19]. Studies that examined associations between African or European BGA using AIMs and adiposity or adiposity-related diseases have reported inconsistent findings with some reporting positive associations with African BGA and adiposity [5, 6, 18, 19] or inverse associations with European ancestry [7], while others reporting inverse associations with African ancestry or no association [13, 14]. For example, a study conducted among African-American and Hispanic-American postmenopausal participants in the Women’s Health Initiative (WHI) found significant positive associations between African ancestry and BMI in the overall population, as well as in the admixed African-American and Hispanic-American subgroups [6]. However, when waist-to-hip ratio (WHR) was used as the measure of adiposity, a significant positive association with African ancestry was attenuated in the overall population, and not observed among African-Americans or Hispanic-Americans. Interestingly, Amerindian ancestry was positively associated with WHR but not with BMI in the overall population and among the Hispanic-American participants in this cohort [6]. Associations between African ancestry and type II diabetes, a disease closely tied to obesity, were also observed among African-American participants in this study [5]. However, a study conducted among elderly Puerto Ricans in the U.S. found negative associations between African genetic ancestry and type II diabetes and cardiovascular disease [13]. Another study found significant positive correlation between obesity/BMI and European ancestry rather than African ancestry [14].

Given these contradictory findings, we sought to further evaluate the relationship between BGA and adiposity using different measures of adiposity, i.e., BMI, percent body fat (PBF), and WHR, including changes in these measures over time, in the Boston Area Community Health (BACH) Survey. A previous study performed on the BACH I cohort (2000–2002) found a positive association between African-American race and obesity as well as significant effect-modification of this association by gender [20]. Building on this research, we sought to 1) to evaluate the relationship between BGA and adiposity (as measured by BMI, WHR, and PBF) and change in adiposity over time, and 2) to evaluate whether these associations are modified by gender, age, diet, physical activity, income, and educational level. As previous research indicates that body fat distribution may not be uniform across ethnic groups and BMI may not provide accurate measures of adiposity for all individuals [15, 21–23], we evaluated associations between genetic ancestry and different measures of adiposity i.e., BMI, WHR, and PBF.

## Methods

### Study design, participants and data collection

The Boston Area Community Health (BACH) Survey is a population-based prospective cohort study that has recruited approximately equal proportions of non-Hispanic white, non-Hispanic black, and Hispanic-American participants from Boston, MA, using multistage stratified cluster sampling. As detailed elsewhere [24, 25], this survey was conducted in three waves that have spanned over a period of ten years (2002–2012). During the first wave of the study (BACH I) (2002–2005), investigators recruited 5,502 men and women aged 30–79 years. The second (BACH II) and third waves (BACH III) of the study were conducted in 2008–10 and 2010–12, respectively. Of the initial 5,502 participants, 3,155 participated in BACH III.

During all three waves, study participants were interviewed in their homes in English or Spanish, and anthropomorphic measurements including height, weight, waist circumference, and hip circumference were taken by trained interviewers. PBF was also assessed during BACH II and BACH III. In addition, data on multiple socio-economic and behavioral factors, co-morbidities, and medication use were collected. During BACH III, investigators also obtained blood samples from participants to determine the proportion of allelic markers. Of the 3,155 participants who participated in the third follow-up survey (BACH III), we excluded participants who reported weight loss in the absence of decreased food intake or increased exercise (n = 409), who were pregnant (n = 1), had diagnosed or undiagnosed diabetes (n = 980), ever had congestive heart failure (n = 142) at the BACH III survey, or reported ever having had a diagnosis of AIDS (n = 19), cancer (n = 283), chronic lung disease (n = 235), or Alzheimer’s disease (n = 12) at the BACH I survey, as these conditions could result in weight change. Even though we were unable to exclude individuals with end-stage renal disease due to lack of information, few, if any, such individuals would have been sufficiently healthy to have been recruited to the BACH study. We were left with 1,726 participants for the analyses. Of these, 654 were self-identified non-Hispanic white participants, while 531 and 541 were self-identified black and Hispanic-Americans, respectively. All participants provided written informed consent. This study was approved by the New England Research Institutes’ Institutional Review Board.

### Measurement of AIMs and BGA

A total of 63 allelic markers, i.e., single nucleotide polymorphisms (SNPs) selected on their ability to estimate percent West-African and Native-American BGA, were genotyped by the Genetic Analysis Platform at the Broad Institute in Cambridge, MA using iPLEX (Sequenom). Ancestry percentages were estimated for each participant using ADMIXTURE software (v. 1.12 http://www.genetics.ucla.edu/software/admixture/).

### Outcome measurement

At BACH I and III, BMI was calculated by dividing weight (kg) by height squared (m^2^). WHR was calculated by dividing waist circumference (cm) by hip circumference (cm). At BACH II and III PBF was measured via bioelectrical impedance using the Tanita scale. We calculated the percent changes in BMI and WHR between waves I and III of the BACH Survey over a mean duration of approximately seven years, and percent changes in PBF between waves II and III over a mean duration of approximately 2.5 years.

### Measurement of covariates

Data on socio-economic and behavioral factors for this analysis were obtained from the BACH III assessment. For socio-economic variables, we grouped participants into categories of **annual household income** (<$20 000, $20 000 - $49 999, and ≥$50 000), **occupation** (office/professional/management, service professions, manual labor, and never worked), and **education** (college or higher, some college or Associate’s degree, high school completed, and less than high school) using cut-offs based on prior studies [26].

In order to assess each participant’s **nutritional intake**, we administered the Block Food Frequency Questionnaire (FFQ) in English or Spanish, and rated each participant’s dietary intake on a scale from 0 to 7. The FFQ Score was determined by summing up points assigned to participants based on the average daily intake of sodium (1 point if <1 500g; 0 otherwise), fiber (1 point if between 25-30g; 0 otherwise), saturated fat (1 point if <14g; 0 otherwise), and the average number of daily servings of vegetables (1 point if between 3–4; 0 otherwise), fruits (1 point if between 2–3; 0 otherwise), meat (1 point if between 2–3; 0 otherwise), and grain (1 point if between 6–11; 0 otherwise). The cutoff values were based on United States Department of Agriculture [27] and American Heart Association guidelines for healthy eating [28]. As most participants’ food frequency scores were low, we dichotomized FFQ scores at a score of 2, with scores above 2 indicating healthier eating.

We measured **physical activity** by using the Physical Activity Scale for the Elderly (PHASE) [29]. This 12 item questionnaire assessed each participant’s time spent engaging in leisure, household, and occupational activities, and walking and sports during the prior week. The hours spent in each activity was weighted by the estimated amount of energy spent. We summed these values over all activities to obtain a PHASE score and categorized this score as low (<100), medium (100–249), or high (≥250).

### Statistical Analyses

In order to minimize potential biases and reduction in precision due to missing data, we performed multiple imputations using Multivariate Imputation by Chained Equations (MICE) [30] in R (R Foundation for Statistical Computing, Vienna, Austria) to create 15 datasets for each combination of race and gender.

The analyses were performed using SUDAAN 11 (Research Triangle Park, NC). The data were weighted by the inverse of the probability of being sampled at baseline (BACH I) to account for oversampling of minority groups. We observed greater attrition among men and participants in the lower socio-economic strata. Thus, the analyses were also adjusted for non-response and loss-to-follow-up using the propensity cell adjustment approach [31], and post-stratified to the Boston census population in 2010.

We assessed all outcomes per 10% rather than 1% greater proportion of genetic ancestry to avoid reporting small effect-estimates. We log-transformed cross-sectional BMI and presented the effects as a percent difference per 10% greater proportion of BGA, as the distribution of its residuals deviated from normality. All other outcome residuals were normally distributed. We performed univariable and multivariable linear regressions to assess outcomes associated with West-African and Native-American ancestry. We also evaluated for effect-modification by age, gender, diet, physical activity, income, and education by including interaction terms between these variables and the exposures in separate models.

Statistical significance for all analyses was initially set at *p* <0.05 using a two-tailed test. In order to account for multiple comparison complexities resulting from examining six different adiposity outcomes in the overall population and different sub-populations, we also assessed our results applying more stringent criteria of *p*<0.008 for statistical significance (i.e., alpha’ = 0.05/6).

## Results

Table 1 provides demographic, socio-economic, behavioral, and outcome data for the overall population, as well as for non-Hispanic white, non-Hispanic black, and Hispanic-American subgroups. The weighted proportions of European, West-African and Native-American BGA in the overall population were 64.6%, 28.1%, and 7.3%, respectively. Self-identified whites had a higher proportion of European allelic markers (86.4%) than West-African (8.5%) or Native-American (5.4%) ancestral markers. Among self-identified blacks, 78.1% of allelic markers were West-African, while 16.6% and 5.3% were European and Native-American, respectively. Among Hispanic-Americans, 48.7%, 29.6%, and 21.7% of the AIMs were European, West-African, and Native-American, respectively.

**Table 1:** Characteristics of the overall population and by self-identified race/ethnicity.

	Overall population	White	Black	Hispanic
	N = 1,726^a^	N = 654 ^a^	N = 531 ^a^	N = 541 ^a^
**Percentage BGA**				
**Mean** ^b^ **(95% CI)**				
West-African	28.10 (25.11 – 31.10)	8.51 (7.44 – 9.58)	78.08 (75.29 – 80.88)	29.64 (25.41 – 33.88)
Native-American	7.30 (6.55 – 8.04)	5.14 (4.45 – 5.83)	5.27 (4.29 – 6.25)	21.69 (17.68 – 25.70)
European	64.60 (61.55 – 67.64)	86.35 (85.05 – 87.65)	16.64 (14.00 – 19.29)	48.66 (43.64 – 53.69)
**Age category**				
**N (%)** ^b^				
34 – 44 yrs.	403 (43.69)	136 (40.33)	115 (45.05)	151 (57.50)
45 – 54 yrs.	564 (27.09)	165 (25.35)	200 (30.77)	199 (28.66)
55 – 64 yrs.	437 (14.76)	185 (16.15)	122 (14.00)	129 (6.91)
65 – 74 yrs.	223 (8.67)	106 (10.49)	70 (6.91)	47 (3.07)
75+ yrs.	100 (5.79)	62 (7.68)	24 (3.26)	15 (1.34)
**Gender**				
**N (%)** ^b^				
Male	643 (46.19)	278 (47.81)	186 (41.94)	179 (46.27)
Female	1083 (53.81)	376 (52.19)	345 (58.06)	362 (53.73)
**Income category**				
**N (%)** ^b^				
<20,000	561 (20.07)	123 (14.40)	173 (27.24)	265 (34.11)
20,000–54,000	542 (25.19)	144 (17.36)	200 (37.93)	198 (39.49)
55,000+	623 (54.73)	387 (68.24)	158 (34.73)	78 (26.40)
**Occupation**				
**N (%)** ^b^				
Professional, Managerial, Sales and Office work	941 (68.74)	489 (78.49)	288 (58.11)	164 (41.06)
Service	453 (17.48)	83 (11.99)	142 (24.72)	227 (30.66)
Manual labor	238 (10.60)	63 (7.41)	87 (15.74)	88 (16.50)
Never worked	94 (3.18)	19 (2.11)	13 (1.42)	61 (11.78)
**Education**				
**N (%)** ^b^				
Less than High school	239 (5.50)	24 (1.71)	60 (5.98)	156 (23.21)
High school or equivalent	490 (22.21)	111 (14.11)	187 (35.92)	191 (35.99)
Some college or Associates degree	372 (19.16)	103 (12.89)	156 (34.91)	113 (20.11)
College or advanced degree	625 (53.12)	416 (71.29)	128 (23.19)	81 (20.70)
**Healthy Eating Score**				
**N (%)** ^b^				
**(N = 1193** ^**c**^ **)**				
Low	932 (75.77)	395 (71.61)	263 (83.56)	274 (85.48)
Relatively High	261 (24.45)	146 (28.39)	63 (16.44)	52 (14.52)
**Mean caloric intake (95% CI) (log transformed kilocalories)** ^b^	7.37 (7.33, 7.41)	7.42 (7.37, 7.46)	7.33 (7.24, 7.42)	7.19 (7.10, 7.29)
**Physical Activity**				
**N (%)** ^b^				
Low	516 (25.57)	194 (26.03)	168 (25.58)	154 (23.34)
Medium	911 (54.73)	351 (55.30)	268 (52.51)	292 (56.11)
High	298 (19.70)	109 (18.67)	95 (21.91)	94 (20.55)
**Baseline BMI** (BACH I)				
**N (%)** ^b^				
Normal (<25 kg/m^2^)	478 (27.85)	241 (38.53)	116 (23.38)	121 (30.79)
Overweight (25-<30kg/m^2^)	665 (39.02)	244 (39.73)	173 (30.49)	247 (40.06)
≥30 kg/m^2^)	583 (33.13)	169 (21.74)	242 (46.13)	172 (29.15)
**Current BMI** (BACH III)				
**N (%)** ^b^				
Normal (<25 kg/m^2^)	403 (33.86)	213 (32.86)	99 (19.39)	91 (19.31)
Overweight (25-<30 kg/m^2^)	660 (37.53)	248 (40.00)	192 (33.94)	219 (43.80)
Obese (≥30 kg/m^2^)	663 (28.61)	194 (27.14)	239 (46.67)	230 (36.89)
**BMI (kg/m** ^**2**^ **)**				
Median (p25, p75)	28.55 (25.25, 32.40)	27.09 (23.87, 30.70)	29.24 (26.00, 33.89)	29.29 (26.37, 32.69)
**Percent change in BMI** ^**d**^				
Median (p25, p75)	1.68 (-3.68, 8.21)	0.78 (-3.90, 7.08)	0.85 (-4.78, 7.26)	3.47 (-1.86, 10.97)
**Absolute change in BMI** ^**d**^				
Median (p25, p75)	0.46 (-1.00. 2.20)	0.22 (-1.04, 1.83)	0.25 (-1.37, 2.14)	1.03 (-0.54, 3.02)
**WHR**				
Median (p25, p75)	0.90 (0.83, 0.95)	0.89 (0.82, 0.95)	0.90 (0.84, 0.95)	0.90 (0.84, 0.96)
**Percent change in WHR** ^**d**^				
Median (p25, p75)	3.98 (-0.46, 9.29)	3.73 (-0.25, 8.68)	3.55 (-0.88, 8.67)	4.45 (0.02, 10.54)
**Absolute change in WHR** ^**d**^				
Median (p25, p75)	0.03 (-0.00, 0.08)	0.03 (-0.00, 0.07)	0.03 (-0.01, 0.07)	0.04 (0.00, 0.09)
**PBF**				
Median (p25, p75)	35.00 (29.00, 41.00)	33.00 (26.00, 39.00)	38.00 (30.00, 43.00)	36.00 (30.00, 40.00)
**Percent change in PBF** ^**e**^				
Median (p25, p75)	0.00 (-8.33, 9.76)	0.00 (-9.38, 8.82)	0.00 (-8.16, 10.00)	2.08 (-7.32, 10.34)
**Absolute change in PBF** ^**e**^				
Median (p25, p75)	0.00 (-3.00, 3.00)	0.00 (-3.00, 3.00)	0.00 (-3.00, 3.00)	1.00 (-3.00, 3.00)

^*a*^ *Mean sample size for 15 datasets; total counts may not always add up as numbers were not the same for all 15 data sets (the number deleted was based on imputed values for each data set); the percentages may not add up to 100% due to rounding;*

^*b*^ *means and percentages are weighted;*

^*c*^ *data not available for 533 participants;*

^*d*^ *between BACH I and III*,

^*e*^ *between BACH II and III; CI = confidence interval; p25 = lower quartile; p75 = upper quartile*.

At BACH I, 76.6% and 69.2% of blacks and Hispanics were overweight or obese, respectively, compared to 61.5% of whites. A greater proportion of Hispanics compared to white or black participants moved from normal to overweight/obese BMI categories between BACH I and III, with 80.7%, 80.6%, and 67.1% of Hispanic, black, and white participants overweight or obese at BACH III. In the unweighted cross-sectional data, we observed a higher median BMI (29 kg/m^2^ vs. 27 kg/m^2^), PBF (36–38% vs. 33%), and slightly higher WHR (0.90 vs. 0.89) among African-Americans and Hispanic-Americans compared to non-Hispanic whites. Hispanic participants had larger increases in all longitudinal measures of adiposity (median percentage change in BMI, WHR and PBF: 3.5%, 4.5% and 2%) than did African-American or white participants, who had approximately equal median percent increases in BMI (0.8–0.9%) and WHR (3.6–3.7%), and no increase in PBF.

### Cross-sectional measures of adiposity

Table 2 presents the results of the cross-sectional analyses. We observed 1.10% higher BMI for each 10% greater proportion of West-African ancestry in the unadjusted analyses (*p*<0.0005). The strength and significance of this association remained after adjusting for age and gender. However, additionally adjusting for socio-economic and behavioral variables reduced the difference to 0.62% (*p* = 0.04), which failed to reach the level of statistical significance required to account for multiple comparisons (*p*<0.008). This reduction was mostly due to adjustment for educational level and income. We observed no association between BMI and Native-American ancestry. In the multivariable analyses for cross-sectional BMI, approximately 8% of the total variance was attributable to West-African ancestry and approximately 8% to Native-American ancestry.

**Table 2:** Associations between genetic ancestry (per 10% greater proportion of BGA) and BMI, WHR and PBF (N = 1726).

	BMI	*p*-value	WHR	*p*-value	PBF	*p*-value
Percent difference (95% CI)	β (95% CI)	β (95% CI)
**Model 1** ^**a**^						
European	0.00		0.00		0.00	
West-African	1.10 (0.61, 1.58)	<0.001^**^	0.0001 (-0.0018, 0.0020)	0.89	0.52 (0.29, 0.75)	<0.001^**^
Native-American	0.84 (-0.16, 1.85)	0.1	0.0057 (0.0012, 0.0102)	0.01	0.08 (-0.34, 0.49)	0.72
**Model 2** ^**b**^						
European	0.00		0.00		0.00	
West-African	1.10 (0.60, 1.58)	<0.001^**^	0.0011 (-0.0007, 0.0029)	0.23	0.50 (0.30, 0.70)	<0.001^**^
Native-American	0.82 (-0.19, 1. 84)	0.11	0.0054 (0.0016, 0.0091)	<0.01	0.27 (-0.13, 0.66)	0.19
**Model 3** ^**c**^						
European	0.00		0.00		0.00	
West-African	0. 62 (0.04, 1. 20)^d^	0.04	-0.0004 (-0.0026, 0.0018)	0.73	0.35 (0.11, 0.58)^e^	<0.01
Native-American	-0.63 (-2.01, 0.77)	0.38	0.0006 (-0.0046, 0.0057)	0.83	-0.15 (-0.72, 0.42)	0.61

^*a*^ *Univariate analysis;*

^*b*^ *Adjusted for age and gender only;*

^*c*^ *Adjusted for age*, *gender*, *income*, *education*, *healthy eating score*, *physical activity*, *caloric intake*, *and occupation; CI = confidence interval; β = effect estimate for log BMI*, *or WHR or PBF;*

^*d*^ *44% decrease in effect estimate was mostly due to adjustment for educational level and income;*

^*e*^ *30% decrease in effect estimate was mostly due to adjustment for educational level;*

*significant at p<0.*005;*

**significant at p<0.*0005*.

We also observed positive associations between PBF and West-African ancestry in the unadjusted and adjusted analyses (0.52 and 0.35 higher PBF, respectively, per 10% greater proportion of West-African ancestry). The attenuation of the effect estimate in the multivariable analysis was mostly due to adjustment for educational level which was inversely associated with the outcome (*p*<0.004). No associations were observed between Native-American ancestry and PBF in the unadjusted or adjusted analyses.

WHR was not associated with West-African ancestry in the univariable or multivariable analyses. We observed statistically significant positive associations between Native-American ancestry and WHR in the unadjusted and age and gender-adjusted analyses. However, these associations no longer remained after adjustment for socio-economic and behavioral variables.

Among socio-economic and behavioral variables associated with higher cross-sectional measures of adiposity, we observed significant inverse associations with higher educational status for BMI (*p*<0.01) (Table 3) and PBF (*p*<0.004). Of note, West-African and Native-American BGA were inversely associated with a higher educational level (OR for college or higher education vs. less for West-African ancestry: 0.75, 95% CI: 0.71, 0.80, *p* <0.0001; for Native-American ancestry: 0.72, 95% CI: 0.63, 0.82, *p* <0.0001) and higher income (OR for income ≥ $50,000 vs. less for West-African ancestry: 0.84, 95% CI: 0.79, 0.88, p<0.0001; for Native-American ancestry: 0.75, 95% CI: 0.65, 0.86, p<0.0001) in this study population.

**Table 3:** Multivariable results for cross-sectional BMI (N = 1726).

	Percent difference in BMI^*^ (95% CI)	*p*-value
**BGA**		
West-African	0. 62 (0.04, 1. 20)	0.04
Native-American	-0.63 (-2.01, 0.77)	0.38
European	0.00	
**Age category**		
34 – 44 yrs.	1.97 (-4.11, 8.44)	0.53
45 – 54 yrs.	3.83 (-2.10, 10.13)	0.21
55 – 64 yrs.	4.83 (-0.88, 10.86)	0.10
65 – 74 yrs.	2.02 (-3.78, 8.16)	0.50
75+ yrs.	0.00	
**Gender**		
Male	1.01 (-1.98, 4.08)	0.56
Female	0.00	
**Income category**		
<20,000	4.32 (-0.69, 9.58)	0.09
20,000–54,000	2.09 (-2.06, 6.43)	0.33
55,000+	0.00	
**Occupation**		
Professional, Managerial, Sales and Office work	-4.24 (-10.38, 2.34)	0.20
Service	-3.54 (-10.24, 3.67)	0.33
Manual labor	-4.89 (-11.74, 2.50)	0.19
Never worked	0.00	
**Education**		
Less than high school	4.54 (-2.59, 12.19)	0.22
High school or equivalent	6.80 (1.43, 12.47)	0.01
Some college or Associates degree	7.04 (2.41, 11.91)	0.00
College or advanced degree	0.00	
**Healthy Eating Score**		
Low	0.06 (-3.45, 3.69)	0.98
Relatively High	0.00	
**Physical Activity**		
Low	2.26 (-2.42, 7.14)	0.35
Medium	1.53 (-2.25, 5.46)	0.43
High	0.00	

*Models adjusted for age, *gender*, *income*, *education*, *healthy eating score*, *physical activity*, *caloric intake*, *occupation*, *and ancestry*.

### Longitudinal measures of adiposity

Table 4 provides associations between BGA and longitudinal measures of adiposity. We observed no association between genetic ancestry and percent change in BMI or WHR between surveys performed at BACH I and BACH III, or percent change in PBF between BACH II and BACH III. Neither income nor educational level was associated with the longitudinal measures of adiposity in the multivariable analyses (S1 Table). However, for longitudinal BMI, we observed a protective effect among employed individuals as compared to those who never worked, regardless of the type of occupation.

**Table 4:** Associations between genetic ancestry (per 10% greater proportion of BGA) and percent change in adiposity (N = 1726).

	Percent change in BMI^d^	*p*-value	Percent change in WHR^d^	*p*-value	Percent change in PBF^e^	*p*-value
β(95% CI)	β (95% CI)	β (95% CI)
**Model 1** ^**a**^						
European	0.00		0.00		0.00	
West-African	0.07 (-0.19, 0.33)	0.61	-0.18 (-0.36, 0.01)	0.06	0.48 (-0.27, 1.23)	0.21
Native-American	0.49 (-0.26, 1.24)	0.2	0.01 (-0.42, 0.44)	0.97	0.30 (-1.47, 2.08)	0.74
**Model 2** ^**b**^						
European	0.00		0.00		0.00	
West-African	-0.00 (-0.26, 0.26)	0.98	-0.19 (-0.37, -0.01)	0.04	0.48 (-0.28, 1.23)	0.21
Native-American	0.36 (-0.37, 1.09)	0.33	0.04 (- 0.37, 0.45)	0.85	0.13 (-1.65, 1.91)	0.88
**Model 3** ^**c**^						
European	0.00		0.00		0.00	
West-African	-0.06 (-0.37, 0.25)	0.72	-0.13 (-0.34, 0.08)	0.22	0.43 (-0.51, 1.37)	0.37
Native-American	-0.13 (-0.99, 0.73)	0.77	0.04 (-0.52, 0.61)	0.88	-0.07 (-2.57, 2.42)	0.95

^*a*^ *Univariate analysis;*

^*b*^ *Adjusted for age and gender only;*

^*c*^ *Adjusted for age*, *gender*, *income*, *education*, *healthy eating score*, *physical activity*, *caloric intake*, *and occupation; CI = confidence interval;*

^*d*^ *between BACH I and III;*

^*e*^ *between BACH II and III*.

The strengths of positive associations were somewhat diminished when analyses were repeated in the entire cohort without applying exclusion criteria. However, the directionality and significance of the results remained unchanged. Similarly, when analyses were repeated including participants with type II diabetes, the effect estimates were slightly diminished but the overall results were essentially unchanged.

### Effect-modification by gender and non-genetic factors

**Fig 1:**
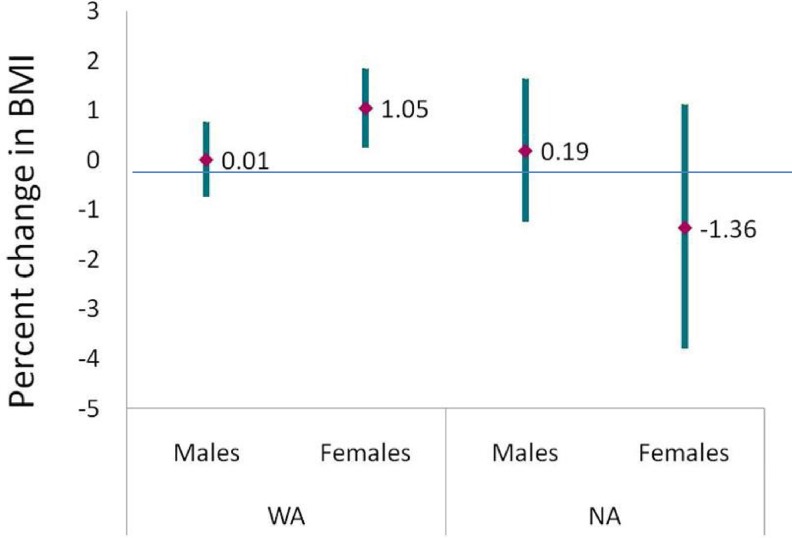
Percent difference in BMI associated with a 10% difference in genetic ancestry by gender. A significant positive association is observed between women of West-African ancestry and BMI (% difference = 0.01; 95% CI: 0.25, 1.84s; *p*<0.01) but not among men of West-African ancestry or men or women of Native-American ancestry. WA = West-African ancestry; NA = Native-American ancestry; CI = confidence interval.

Table 5 provides results for interactions between genetic ancestry and gender and diet. We observed significant effect-modification by gender for the association between West-African ancestry and BMI (*p*-interaction = 0.0019). Similar, albeit less significant, interactions were observed when PBF (*p*-interaction = 0.04) or WHR (*p*-interaction = 0.02) were used as the measure of adiposity. In the analyses stratified by gender (Table 4 and Fig 1), we observed a positive association between West-African genetic ancestry and BMI among women which was significant at the *p*<0.05 level, but not among men. Similarly, the positive association observed between West-African ancestry and PBF among women was substantially attenuated and no longer significant in men. West-African ancestry was not associated with WHR in the stratified analyses.

**Table 5:** Associations between West-African and Native-American genetic ancestry and adiposity, stratified by gender and Healthy Eating Score.

	**BMI % change (95% CI)**			**WHR β (95% CI)**					**PBF β (95% CI)**
	**Males** ^**a**^	**Females** ^**a**^	***p*-int.**	**Males** ^**a**^	**Females** ^**a**^	***p*-int.**	**Males** ^**a**^	**Females** ^**a**^	***p*-int.**
**West-African**	0.01 (-0.74, 0.77)	1.05 (0.25, 1.84)	<0.01^*^	-0.001 (-0.004, 0.002)	0.000 (-0.003, 0.003)	0.02	0.17 (-0.18, 0.52)	0.48 (0.19, 0.77) ^*^	0.04
**Native-American**	0.19 (-1.25, 1.64)	-1.36 (-3.80, 1.13)	0.54	0.004 (-0.002, 0.008)	-0.002 (-0.011, 0.007)	0.79	0.15 (-0.51, 0.81)	-0.37 (-1.26, 0.51)	0.49
	**BMI % change (95% CI)**			**WHR β (95% CI)**					**PBF β (95% CI)**
	**High HE Score** ^**b**^	**Low HE Score** ^**b**^	***p*-int.**	**High HE Score** ^**b**^	**Low HE Score** ^**b**^	***p*-int.**	**High HE Score** ^**b**^	**Low HE Score** ^**b**^	***p*-int.**
**West-African**	0.58 (-0.67, 1. 85)	0.63 (0.00, 1.28)	0.81	-0.002 (-0.007, 0.003)	-0.000 (-0.003, 0.002)	0.44	0.32 (-0.23, 0.87)	0.36 (0.11, 0.61)	0.99
**Native-American**	-4.93 (-8.83, -0.89)	0.78 (-0.71, 2.31)	<0.01^*^	-0.010 (-0.023, 0.004)	0.005 (-0.001, 0.010)	0.01	-1.24 (-2.76, 0.28)	0.26 (-0.37, 0.89)	0.04

^*a*^ *Models additionally adjusted for age*, *income*, *education*, *healthy eating score (HE Score)*, *physical activity*, *caloric intake*, *and occupation;*

^*b*^ *models additionally adjusted for age*, *gender*, *income*, *education*, *physical activity*, *caloric intake*, *and occupation; CI = confidence interval; HE Score = Healthy Eating Score; int*. *= interaction; referent = European Ancestral Markers*: *percent change in BMI = 0*.*0000; WHR = 0*.*0000*, *PBF = 0*.*0000;*

^*^ *significant at p<0*.*005;*

^**^ *significant at p<0*.*0005*.

Given the positive associations we observed between West-African BGA and socio-economic variables, we further examined associations between West-African ancestry and BMI within categories of gender and educational level (college or higher vs. less education) and gender and income (annual income of ≥$50,000 vs. less). We observed a positive association between West-African genetic ancestry and BMI among women without a college degree (BMI percent change = 1.29%, 95% CI: 0.20%, 2.40%), while no such association was observed among women of a higher educational level (BMI percent change = 0.75%, 95% CI: -0.42%, 1.93%) or among men of any educational level (BMI percent change for men with a higher education = 0.28%, 95% CI: -0.99%, 1.56%; BMI percent change for men with less education = -0.36%, 95% CI: -1.35%, 0.63%). Similar patterns were observed for associations between West-African ancestry and PBF for analyses stratified by gender and educational level, and for West-African ancestry and BMI for analyses stratified by gender and income level.

We also observed significant effect-modification by diet for the association between Native-American ancestry and BMI (*p*-interaction = 0.0023). However, interactions between Native-American ancestry and diet were significant only at the *p*<0.05 level when PBF (*p*-interaction = 0.04) or WHR (*p*-interaction = 0.01) were used as the measures of adiposity (Table 5). Among participants with higher Healthy Eating Scores, Native-American ancestry was associated with lower BMI at the *p*<0.05 level of significance. The association remained negative, albeit not statistically significant, when WHR and PBF were used as the measures of adiposity. No negative associations were observed among participants with low Healthy Eating Scores.

We observed no effect-modification between West-African or Native-American genetic ancestry and age, educational level, income, or physical activity for cross-sectional measures of adiposity and no effect modification by any demographic or socio-behavioral variable for longitudinal measures of adiposity.

## Discussion

In this study, we evaluated associations between genetic ancestry and cross-sectional and longitudinal measures of adiposity. We observed positive associations between West-African ancestry and cross-sectional BMI as well as PBF in the unadjusted analyses and after adjusting for multiple socio-economic and behavioral variables, but not with WHR. Thus, our findings suggest that West-African ancestry confers an increased risk for total body adiposity, rather than central adiposity. Contrary to findings of other studies [16, 32], we did not observe significant positive associations between Native-American ancestry and BMI in the unadjusted or multivariable-adjusted analyses. The low prevalence of Native-American ancestral markers in the overall population (7%) may have precluded us from detecting potential associations.

We also observed evidence suggesting possible mediation of associations between genetic ancestry and cross-sectional BMI and PBF by education. The positive associations observed between African ancestry and both BMI and PBF were substantially attenuated after adjusting for educational level. Even though standard techniques cannot be utilized to accurately evaluate mediation in the presence of unmeasured confounding [33], the inverse associations we observed between West-African and Native-American BGA and higher educational level and the inverse associations we observed between higher education and BMI and PBF, further strengthen this possibility.

We did not observe associations between genetic ancestry and percent changes in BMI or WHR over a mean follow-up duration of approximately seven years (median percent change of 1.68% and 3.98% for BMI and WHR, respectively), or with PBF over a mean follow-up duration of approximately 2.5 years (median percent change of 0.00%). However, we are unable to rule out the possibility of detecting such associations over longer periods of follow-up.

We observed evidence of effect modification by gender for associations between West-African ancestry and cross-sectional measures of adiposity with greater risk among women. Furthermore, this effect was restricted to women of a lower socio-economic status. These findings indicate that West-African ancestry confers higher risk of adiposity among women of West-African descent as compared to men of the same ancestral background despite a similar prevalence of adiposity genes across both groups, perhaps due to biological differences between men and women; access to education and other resources may counteract this effect. Given the greater difference in the proportions of African-American women as compared to African-American men in the lower vs. higher income categories (28% vs. -14% greater percentage for women and men, respectively) and lower vs. higher educational categories (17% vs. 7% greater percentage for women and men, respectively), it is also likely that these results reflect the differential distribution of socio-economic variables strongly associated with adiposity among men and women of West-African descent.

These data concur with recent patterns of obesity prevalence in the U.S. The NHANES 2011–2012 Survey report described a substantially greater prevalence of obesity among African-American women compared to African-American men (56.6% vs. 37.1%) [3]. Our results may also partially explain contradictory results observed across studies. The association between African BGA and adiposity may be weakly associated in a population consisting mostly of males, while it may be apparent in a study restricted to women. Of note, the WHI study which found strong positive associations between African ancestry and BMI consisted entirely of women.

We also observed evidence of effect-modification of associations between Native-American ancestry and adiposity by diet despite the relatively low proportion of Native-American ancestral markers in this population. Individuals with a Native-American ancestry who had relatively high Healthy Eating Scores, but not low scores, had negative associations with all cross-sectional measures of adiposity, albeit not all results achieved statistical significance. Further research should investigate the impact of diet on associations between Native-American ancestry and adiposity. The relatively low threshold used (FFQ Score ≥2) in our study to define healthy eating, would suggest that even a small-moderate improvement in diet may be beneficial in this subpopulation.

Our study is not without limitations. First, the BACH study population consisted of no self-identified Native-American participants and thus, had a low prevalence of Native-American AIMs. This may have precluded us from detecting possible associations between Native-American ancestry and adiposity as were observed in other study populations [16, 32]. Second, follow-up durations between BACH I and III (mean duration of approximately seven years), and between BACH II and III (mean duration of approximately 2.5 years) may not have been sufficient to observe possible effects of genetic ancestry on longitudinal measures of adiposity. Finally, due to smaller sample sizes, not all sub-cohort analyses had sufficient power to achieve statistical significance when using the more stringent criteria accounting for multiple comparisons (*p*<0.008).

The strengths of this study include evaluation of multiple measures of adiposity specifically BMI, WHR, and PBF assessed cross-sectionally and over time. While the clinical meaning and utility of the different measures of adiposity likely differ by race/ethnicity, consistent patterns across different measures, as were observed in regard to BMI and PBF among participants of West-African descent in our study population, strengthen the hypothesis that West-African ancestry is associated with increased total body adiposity. In addition, to our knowledge, this was the first study to report on interactions between BGA and non-genetic factors that contribute to adiposity and which could help explain the high prevalence of adiposity in distinct populations.

### Conclusion

We observed elevated risk of overall adiposity among participants with West-African ancestry which was stronger among female participants. Our results also suggested greater protective effects of healthy eating on adiposity for participants of Native-American descent. However, these analyses should be repeated in cohorts with a greater proportion of participants of Native-American BGA in order to confirm our findings. These results also call for the evaluation of longitudinal outcomes of adiposity in cohorts with longer follow-up.

## Supporting Information

S1 Table Multivariable analysis results for longitudinal BMI. This table presents the associations between variables included in the multivariable model and BMI.
(PDF)
