# Supplementary material for: Biogeographic Ancestry Is Associated with Higher Total Body Adiposity among African-American Females: The Boston Area Community Health Survey
Source: PLoS One. 2015 Apr 13;10(4):e0122808. doi: 10.1371/journal.pone.0122808 (PMC4395279; doi:10.1371/journal.pone.0122808)
Supplement: S1 Table — This table presents the associations between variables included in the multivariable model and BMI. (PDF) [file pone.0122808.s001.pdf]

SUPPORTING INFORMATION

Table S1. Multivariable results for percent change BMI between BACH I and III (N = 1726)

	β (95% CI)	*p*-value
**BGA**		
West-African	-0.06 (-0.37, 0.25)	0.72
Native-American	-0.13 (-0.99, 0.73)	0.77
European	0.00	
**Age category**		
34 – 44 yrs.	7.32 (4.40, 10.23)	<0.0001
45 – 54 yrs.	6.49 (3.61, 9.38)	<0.0001
55 – 64 yrs.	3.03 (-0.11, 6.17)	0.06
65 – 74 yrs.	2.10 (-1.19, 5.39)	0.21
75+ yrs.	0.00	
**Gender**		
Male	-0.93 (-3.04, 1.18)	0.39
Female	0.00	
**Income category**		
<20,000	0.95 (-2.66, 4.55)	0.61
20,000-54,000	0.74 (-1.55, 3.02)	0.53
55,000+	0.00	
**Occupation**		
Professional, Managerial, Sales and Office work	-9.20 (-13.73, -4.67)	<0.0001
Service	-7.51 (-12.28, -2.74)	0.002
Manual labor	-9.13 (-14.51, -3.75)	0.0009
Never worked	0.00	
**Education**		
Less than high school	1.15 (-3.39, 5.68)	0.62
High school or equivalent	-0.45 (-3.52, 2.63)	0.78
Some college or Associates degree	1.01 (-1.65, 3.67)	0.46
College or advances degree	0.00	
**Healthy Eating Score**		
Low	0.15 (-1.79, 2.10)	0.88
High	0.00	
**Physical Activity**		
Low	-0.02 (-3.33, 3.29)	0.99
Medium	-0.26 (-2.92, 2.40)	0.85
High	0.00	

[*]Models adjusted for age, sex, income, education, healthy eating score, physical activity, caloric intake, occupation, and ancestry;

CI = confidence interval
